# Optimisation of Ruthenium Dye Sensitised Solar Cells Efficiency via Sn Diffusion into the TiO_2_ Mesoporous Layer

**DOI:** 10.1371/journal.pone.0063923

**Published:** 2013-05-21

**Authors:** Codrin Andrei, Dominic Zerulla

**Affiliations:** 1 Plasmonics and Ultrafast NanoOptics Laboratory, School of Physics, University College Dublin, Dublin, Ireland; 2 Strategic Research Cluster in Solar Energy Conversion, University College Dublin, Dublin, Ireland; Massey University, New Zealand

## Abstract

Dye sensitised solar cells (DSCs) typically include a mesoporous titanium dioxide (TiO_2_) scaffold, sensitised with an adsorbed dye, as the main active element responsible for the photon absorption and charge separation functionalities. The sintering process employed in the TiO_2_ active layer fabrication plays a crucial role in the formation of the nanoparticle (NP) scaffold and hence in the performance of a dye sensitised solar cell, as it allows the particles to form efficient inter-crystalline electric contacts providing high electron conductivity. Furthermore, the DSC design requires a conductive transparent top electrode which is typically made of fluorinated stannic oxide. Here we report on a highly spatially resolved scanning electron microscopy study in conjunction with focussed ion beam milling and energy dispersive X-ray (EDX) mapping of the distribution of all relevant elements within a DSC subsequent to a classical sintering process in the range of 350°C–550°C. Additionally, the article provides quantitative results regarding the found Sn diffusion and its effect on efficiency confirmed via J-V measurements. The effective spatial resolution of the EDX studies was calculated by Monte Carlo simulations of the electron trajectories and X-ray emission region. This permits to construct a model for the migration of Sn from the transparent conductive oxide into the TiO_2_ scaffold, resulting in alterations in the composition of the complex system which has a direct effect on the DSC performance. J-V measurements conclude that sintering temperature of 500°C is close to the optimum regarding Sn diffusion enhancement of DSCs. Sintering temperatures above 500°C were causing a drop in the DSC efficiency and are therefore not recommended. In order to optimize the DSC efficiency, the results are summarized by a model that explains how the efficiency varies with the Sn diffusion process.

## Introduction

Dye sensitised solar cells have been intensively researched and improved since their invention by Michael Grätzel [1] and new efficiency records exceeding 12% have been recently set [2]. The DSC origin can be dated back to the 1960s, when it was shown by Gerischer and Tributsch [3] that photoexcited dye molecules can inject electrons into the conduction band of semiconductor substrates. Further improvements included the adsorption of dye molecules on the surface of the semiconductor [4], [5] and also the use of dispersed particles [6], [7] came as another important feature in the 1980s. Although semiconductors like ZnO sensitised with dyes like cyanine [8] have been tested, titanium dioxide has become the main semiconductor [9] employed in DSCs due to its widely known advantages. Up to 1991, the DSC efficiency was below 1% and the major step was achieved in that year when O’Regan and Grätzel reported a 7.1% efficiency record [1]. This was followed by an increasing number of inorganic dyes [10]-[12] that have been synthesized and multiple organic dyes [13]–[15] with high efficiencies having also been embedded into DSCs. The electrolyte, mostly based on the iodide/triiodide redox mediator (but also recently Co-based electrolytes), has also been continuously improved to optimally match the requirements of each DSC type [2], [16], [17]. Titanium dioxide has remained the most used semiconductor for this technology and has also been continuously researched and improved over the last decade including the TiCl_4_ pre∼ and post∼ treatments [18]. The TiO_2_ sintering process required in the photoanode fabrication plays a vital role in the formation of the nanoparticle scaffold and it directly influences the cell’s quantum efficiency. The sintering allows the nanoparticles to form efficient inter-crystalline electric contacts to provide good electron conductivity. A failure to achieve good electrical contacts automatically translates into low electron conductivity and thus low efficiency. Typical values for the sintering temperature reported in literature are in the range of 450–600 °C. Some of the new directions in DSC research, including low energy manufacturing, usage of flexible plastic electrodes, and integration of metallic nanoparticles [19]–[21], metallic nanostructured substrates [22] or quantum dots [23], require a decrease in the TiO_2_ sintering temperature. Therefore there is a clear demand in understanding how the sintering process will be affected when modifying its parameters and how can the sintering be optimised to yield maximum DSC efficiency.

Ito et al. [24] have shown how the fabrication of DSCs can be optimised and identified the major parameters that need additional research. It is well known that during the sintering process the mesoporous titania layer shrinks substantially, depending on the sintering temperature and duration of sintering. A thick layer of TiO_2_ (e.g. greater than 25 μm) translates into a larger effective area for the dye adsorption (and consequently results in higher light absorption), but will also increase the path length for the electrons/excitons travelling to one of the electrodes and thus will increase the recombination losses and resistivity, resulting in a lower DSC efficiency. A viable compromise is to decrease the thickness of the TiO_2_ mesoporous layer (down to 10 μm or less) in order to decrease the path length for the electrons to travel. As a result, the mean electron diffusion length in a DSC, will be in the range of 10–20 μm [25], thus light can be efficiently harvested to achieve a high incident photon-to-electric current conversion efficiency (IPCE). Although decreasing the TiO_2_ thickness reduces the dark current [26], it also reduces the dye adsorption active area, resulting in a reduced light absorption and consequentially a reduced photocurrent, and this latter effect can be overcome by using for example more highly absorbing dyes or plasmonic enhancement [22]. However, some underlying chemical and physical mechanisms that are at the basis of the photoanode fabrication via the TiO_2_ sintering process, are not yet fully understood. One of them is the TiO_2_ doping process with materials that potentially increase the overall DSC efficiency. It has been reported that the photocatalytic activity of TiO_2_ was increased when using semiconductor substrate-coupled systems like SnO_2/_TiO_2_ [27]–[30]. Duan et al. [31] have reported on using a hydrothermal preparation method to directly achieve Sn doping of TiO_2_ which yields an impressive 12.1% increase in efficiency of a N3 ruthenium sensitised DSC. According to the same authors, the Sn-doping has no effect on the morphology and crystal form of the TiO_2_, but it influences the V_oc_ and the transfer rate of electrons. According to Duan et al. [31], the optimum Sn-doping for a TiO_2_ layer is 0.5% mol. In a comprehensive study on the Sn doping effects on TiO_2_, Xin et al. [32] summarize some of the previous major findings of Do et al. (1994) [30], Lin et al. (1999) [27], Cao et al. (2004) [29] and conclude that doping the anatase lattice with Sn cations enhances the separation of photogenerated pairs. This enhancement is attributed to a reduction in the recombination processes due to coupling of two semiconductors (i.e. TiO_2_ and SnO_2_) with non-equal Fermi levels.

We have already reported that Sn diffusion occurs automatically during the sintering process when using FTO glass electrodes [33]. It is therefore of interest to investigate the potential enhancement of the DSC efficiency using the natural Sn diffusion occurring during the formation of the active layer. An important step to control this effect is the ability to correlate the sintering parameters (e.g. temperature, time) with the induced effects in the DSC efficiency. However, to the best of our knowledge, the effect of the Sn diffusion is not thoroughly investigated over a wide range (350°C –550°C) of sintering temperatures. Thus, this paper investigates the effects of the sintering process on the SnO_2_:F substrate, TiO_2_ mesoporous scaffold and more importantly on the DSC efficiency in the above-mentioned temperature range. Energy dispersive X-ray spectroscopy (EDX) analysis, together with Monte Carlo simulations of the X-ray emission region, have been used in conjunction with scanning electron microscopy (SEM) imaging to spatially resolve the Sn diffusion at various sintering temperatures, and additionally, the correlation of the sintering effects to the current density-voltage (J-V) measurements has been investigated. Therefore, the focus of this study is to investigate if the Sn diffusion during the sintering process can be used in a controlled fashion to optimise the DSC efficiency, and if so, to what extent.

## Materials and Methods

The study was performed in the TiO_2_ sintering temperature range of 350°C –550°C. The lower limit of the TiO_2_ sintering temperature was selected above the amorphous–to–crystalline anatase titanium oxide transition temperature (350°C [34]), while the upper limit of the sintering temperature was selected below the glass transition temperature. The DSC electrodes have been prepared on aluminoborosilicate (AlBoSi) glass slides (*Schott AG*, n = 1.516, 2 cm×2 cm×1.1 mm) that have been coated with a 700 nm (±2%) thick SnO_2_:F layer (10 Ω sq^−1^) by spray pyrolysis. Prior to the TiO_2_ deposition process, the glass slides were subject to a sonication treatment for 30 minutes at room temperature, have been subsequently rinsed with distilled water and ethanol (*Sigma-Aldrich,* 99.8%), and then allowed to dry in a clean sealed container. The photoanodes have been fabricated by depositing TiO_2_ (∼ 2.8 µg paste for each electrode) nanoparticle paste (11% wt. nanocrystalline anatase, with ethanol, organic binders and solvents to adjust for optimum viscosity, *Ti-Nanoxide D* from *Solaronix SA*) on each FTO glass slide (TiO_2_ area 1.69 cm^2^ for each electrode) via the tape casting technique [35]. By using this technique in conjunction with a constant thickness spacer (34.65 µm used for all of the samples), high reproducibility of TiO_2_ final thickness with 2% precision are achieved herein. The final thickness of the layer, after the sintering process, shrinks by a certain factor depending on sintering time and temperature [33]. By keeping the sintering time constant and recording it as a control parameter, the sintering temperature was the varied parameter of this Sn diffusion study. The experiments were made for multiple samples with a high reproducibility, as it will be described furthermore.

**Figure 1:**
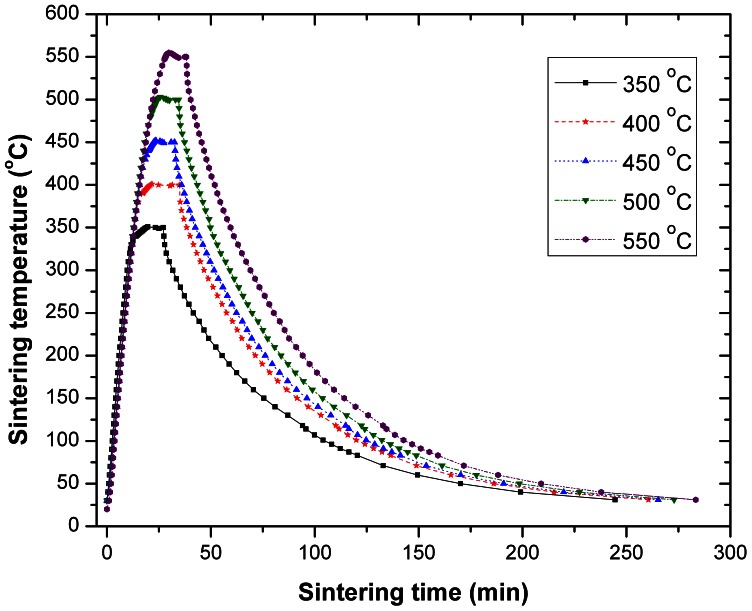
The photoanode sintering temperature as a function of time.

Subsequent to the TiO_2_ paste deposition, the photoanodes have been sintered by programming a temperature controllable oven to ramp-up from room temperature to selected maximum temperatures (in the range of 350–550°C), under normal atmospheric conditions, with a heating rate of 20°C min^−1^. The maximum sintering temperature was kept constant for 20 minutes, followed by a slow, smooth cooling process (Figure 1). The photoanodes have been subsequently post-treated with a TiCl_4_ solution (50 mM, *Sigma-Aldrich,* 99.0%) at 60°C for 20 minutes. Then, the electrodes were thoroughly rinsed with distilled water and re-sintered at 450°C for 20 minutes, followed by the dye adsorption process. A ruthenium N749 solution (0.2 mM) was prepared in ethanol. Subsequently, a 20 mM chenodeoxycholic acid (3α,7α-Dihydroxy-5β-cholanic acid) solution in ethanol has been added as a co-adsorbent. The TiO_2_ electrodes were immersed at an initial temperature of 60°C in the ruthenium N749 dye solution for 24 hours (in a sealed environment and in darkness, and permitted to cool down to room temperature), then thoroughly rinsed with absolute alcohol (*Fluka*, 99.8%) and then finally assembled. The counter electrodes have been fabricated by using identical FTO coated AlBoSi glass slides as described above. A thin layer of hexachloroplatinic acid (5 mM H_2_PtCl_6_ in isopropanol) was deposited on the FTO, followed by a 15 minutes sintering process at 450°C with an average ramp-up rate of 20°C min^−1^. The electrolyte was made of iodide/triiodide (50 mM) redox couple, with pyridine additive and with propionitrile as solvent.

A state of the art *FEI Quanta 3D®* dual beam scanning electron microscope equipped with a highly spatially resolving electron field emission gun, a highly spatially resolving Gallium ion gun and an EDAX Trident XM4 detector, was employed for imaging and depth profiling of the TiO_2_ structures, providing a high spatial resolution in imaging mode of down to 1.5 nm.

## Results and Discussion

The photoanode sintering temperature was recorded as a function of time in the chosen temperature range (350°C –550°C) as a first control parameter (Figure 1). The measurements started from room temperature, up to a maximum temperature that was kept constant for 20 minutes. The slow, smooth cooling process required to prevent the cracking of the electrode, was maintained over a long enough period of time (approx. 2 hours), for each sample from the maximum temperature down to 60°C before removing the photoanode from the oven.

**Figure 2:**
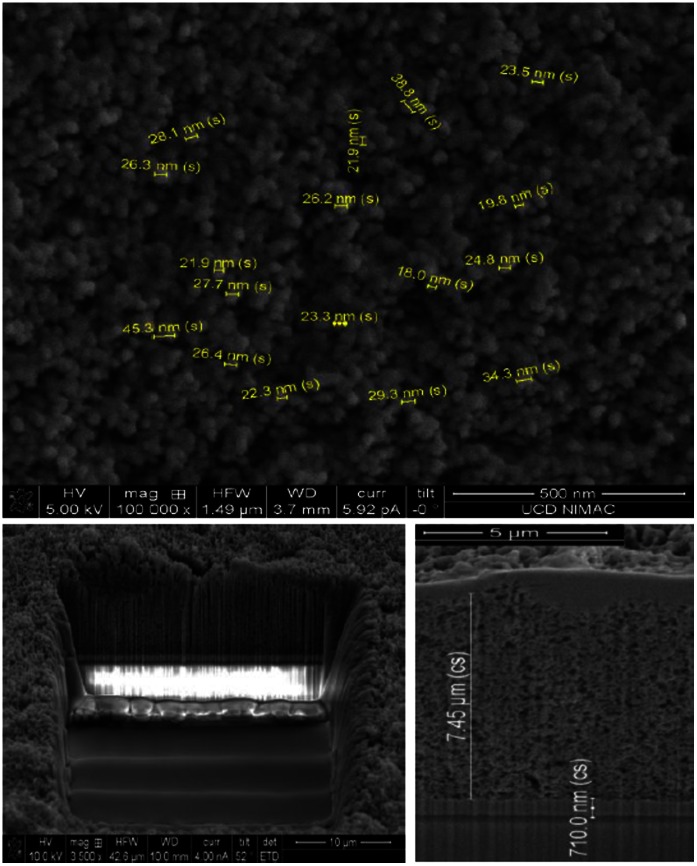
SEM and FIB imaging analysis revealed the average dimensions of the TiO_2_ layer and of the TiO_2_ nanoparticles. **a)** (top) The TiO_2_ nanoparticle average diameter 24 nm; **b)** (bottom left) FIB milling was used to remove a cuboid with dimensions of 20 μm×30 μm×20 μm; **c)** (bottom right) The FTO initial (pre-sintering) thickness 710 nm (averaged cross-section).

The typical TiO_2_ NP size distribution with an average diameter of 24 nm for the TiO_2_ nanoparticles is exemplified in Figure 2a using FIB imaging. By analysing all of the samples with the FIB imaging technique, it was concluded that the TiO_2_ mesoporous layer surface was reasonably homogeneous and therefore the tape casting technique remained the preferred deposition technique. In order to evaluate the absolute thicknesses of the TiO_2_ scaffold and of the FTO layer, technique focused ion beam (Ga) was used to remove a cuboid with dimensions of 20 μm×30 μm×20 μm (Figure 2b). The FIB milling provides access to the buried layer structure and allows precise thickness measurements of the TiO_2_, FTO layers to be recorded. The FTO cross-sectional height of photoanode samples prior to the sintering process (containing a layer of TiO_2_ as a reference layer) was measured via FIB imaging to have an average value of 710 nm (Figure 2c).

**Figure 3:**
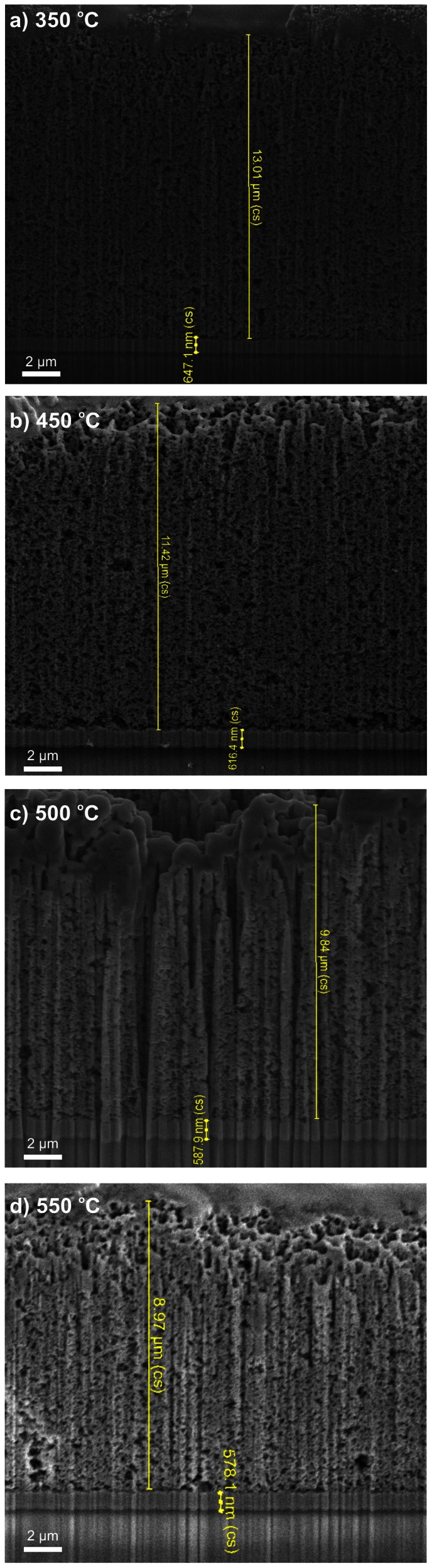
FIB imaging measurements of TiO_2_ and SnO_2_:F cross-sections after sintering at a) 350°C; b) 450°C; c) 500°C; d) 550°C.

Subsequent to the FIB milling, ion imaging revealed that the TiO_2_ thickness decreased as expected with increasing sintering temperatures, from 13.01 µm (photoanodes sintered at a maximum temperature of 350°C, Figure 3a), to 11.42 µm (450°C, Figure 3b), down to 9.84 µm (500°C, Figure 3c) and 8.97 µm (550°C, Figure 3d) respectively. The next layer that can be clearly identified in Figure 3 by its different structure and secondary electrons brightness is the SnO_2_:F layer. The FTO’s thickness was also determined to decrease when increasing the sintering temperature, for the used duration of 20 minutes sintering in the range of 350°C –550°C. As measured here it decreases from 647 nm (Figure 3a), to 616 nm (Figure 3b), down to 587 nm (Figure 3c) and 578 nm (Figure 3d) respectively.

**Figure 4:**
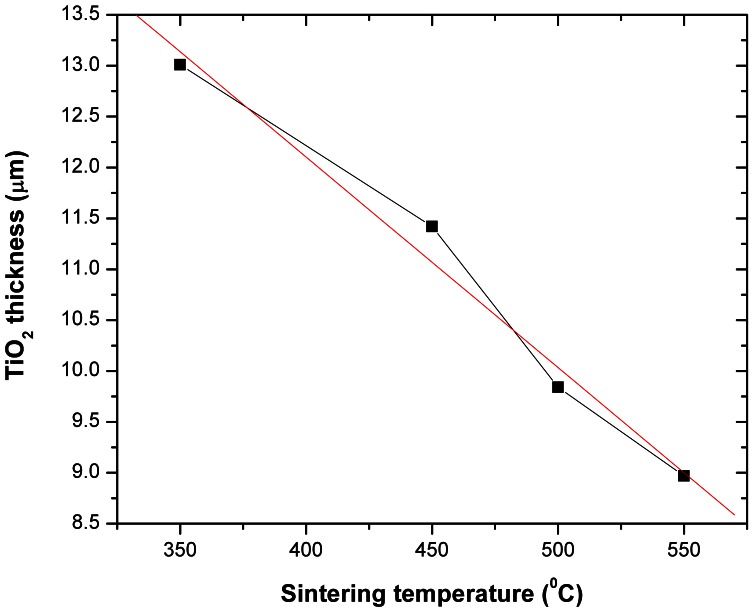
The TiO_2_ thickness as a function of the sintering temperature for constant maximum sintering temperature times of 20 minutes.

**Figure 5:**
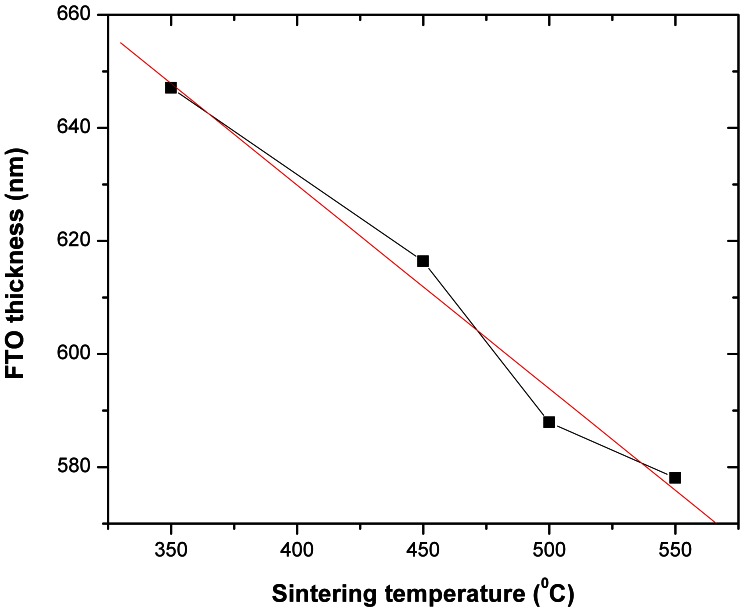
The FTO thickness as a function of the sintering temperature, showing a decrease between 350°C –550°C.

The next figures correlate the TiO_2_ thickness to the sintering temperature (Figure 4), and the FTO thickness to the sintering temperature (Figure 5) for identical duration of sintering at the maximum temperature. The TiO_2_ thickness as a function of the sintering temperature (for constant max. temperature durations of 20 minutes) in the 350°C –550°C range can also be used as a first approximation method for the TiO_2_ final thickness, prior to ellipsometry measurements or alternatively FIB/SEM cross-section determinations.

In an earlier paper [33] we found first evidence of the diffusion of the FTO layer during the sintering process, into the TiO_2_ scaffold but not into the glass substrate. The FTO thickness from the images shown in Figure 3, is analysed in Figure 5 as a function of the sintering temperature. It is concluded that the FTO thickness decreases with increasing sintering temperature for the selected sintering duration and in the studied range of 350°C –550°C.

**Figure 6:**
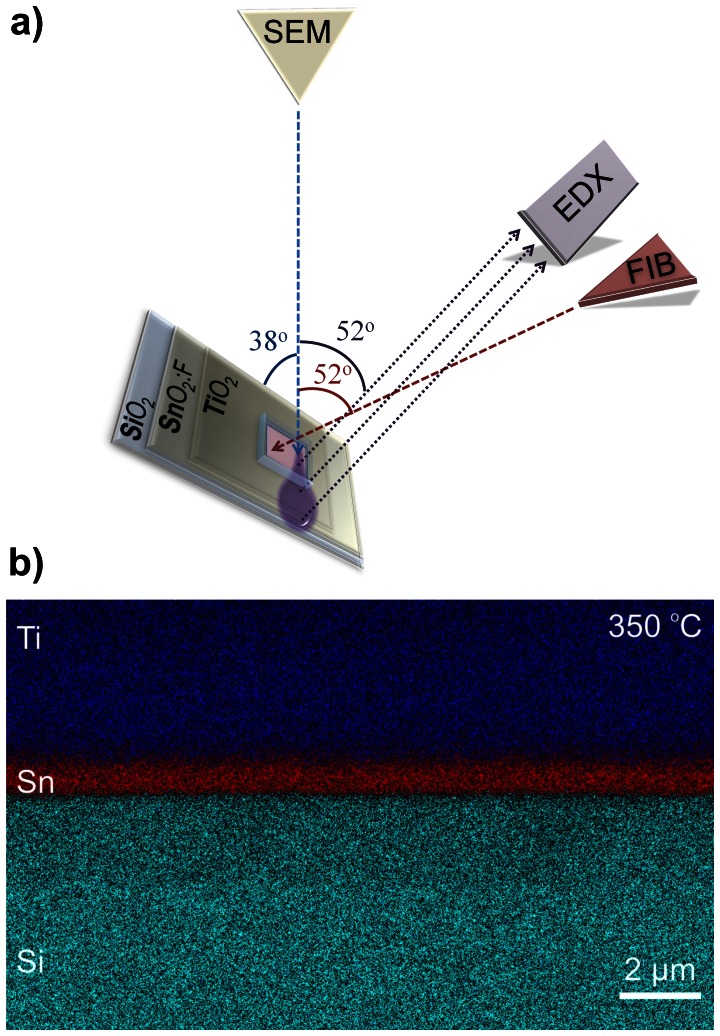
The schematic of the EDX setup geometry (a) and an example of the EDX analysis (b) used to reveal the elemental layer composition (Ti, Sn, Si) for the sintering temperature range of 350–550°C.

**Figure 7:**
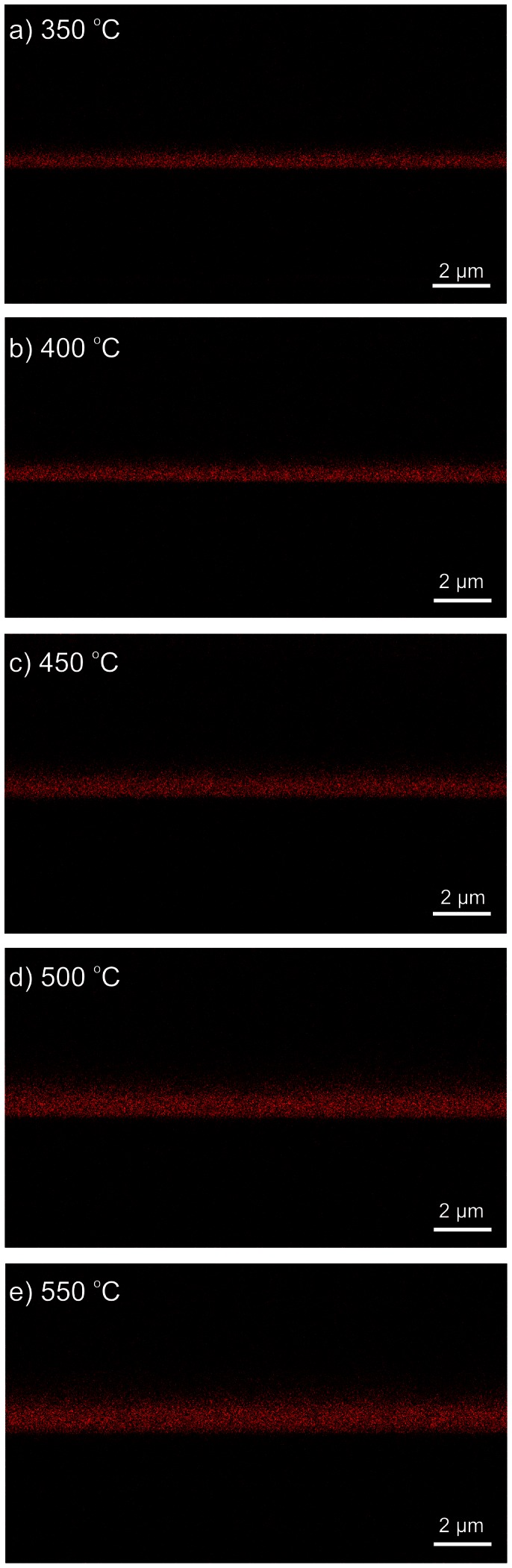
The EDX analysis on the Sn diffusion for samples sintered at: a) 350°C; b) 400°C; c) 450°C; d) 500°C; e) 550°C.

**Figure 8:**
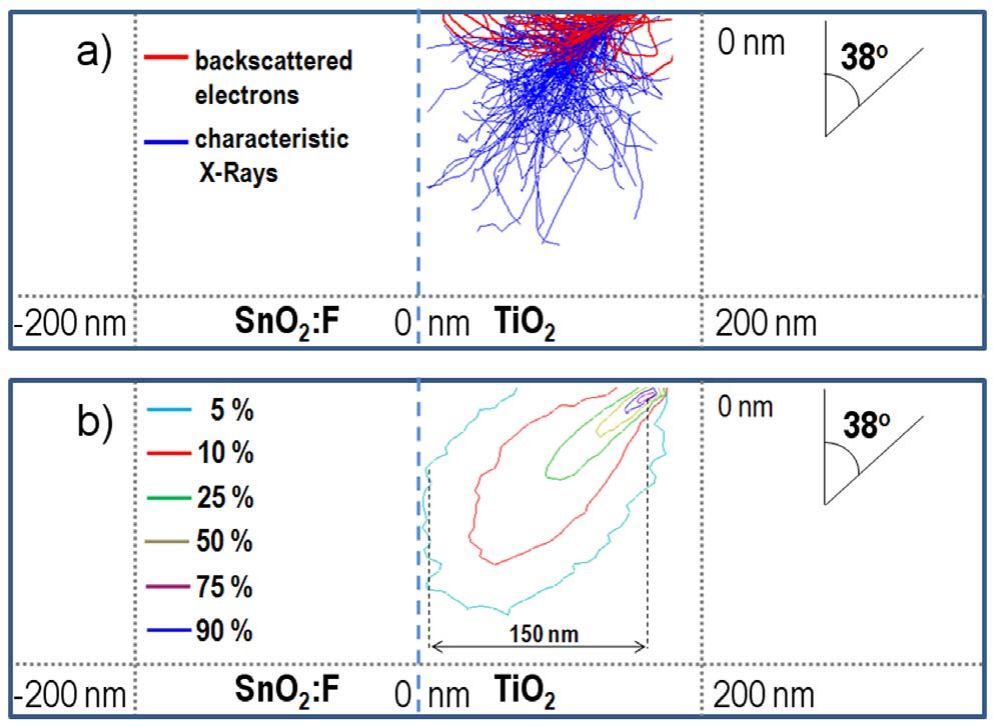
Simulations of a) electrons trajectories and b) X-Ray emission region in the EDX analysis. Incident electron plume determined to be approx. 150 nm for an accelerating voltage of 10 keV that was used experimentally.

While the decrease in the measured thickness for the mesoporous TiO_2_ layer can be attributed to the necking process which reduces the average spacing between the NPs, the decrease in the FTO cross-section can be attributed to the FTO diffusion. Unsurprisingly, the FTO is diffusing with higher rates when sintered at higher temperatures. To confirm and quantify the diffusion effect in the above-mentioned temperature range, EDX spatial mapping was employed for samples sintered at 350°C, 400°C, 450°C, 500°C and 550°C. The setup geometry is shown in Figure 6 with the EDX detector and the FIB gun oriented at 52° from the SEM electron gun (and normal to the sample) and consequently with the sample tilted accordingly at 52° in respect to the electron beam. In the EDX analysis, the electron beam was kept at 10 keV for all the samples to achieve an electron volume interaction depth (violet plume in Figure 6a) below the micrometer range (i.e. to gather signals from the TiO_2_, SnO_2_ down to the glass). Multiple scattering Monte Carlo simulations of the electron trajectories and the X-ray emission region have been carried out in Casino v2 [36], to determine the spatial extent of the 3-D X-ray emission region from which the EDX detector has received its spectral information. Based on these simulations, experimental EDX data as shown furthermore in Figure 7 was collected for multiple samples sintered in the studied range of 350°C –550°C, using the 10 keV acceleration voltage. Figure 8a shows the electron trajectories, for 200.000 simulated electrons incoming with an acceleration voltage of 10 keV, an incident beam radius of 4 nm at 38° with respect to the photoanode surface. The extent of the electron plume and the X-ray emission region, simulated in Figure 8b **,** was measured to be within 150 nm for a minimum remaining electron energy of 3.443 keV (which corresponds to the energy of the Sn principal emission line, Lα_1_).

**Figure 9:**
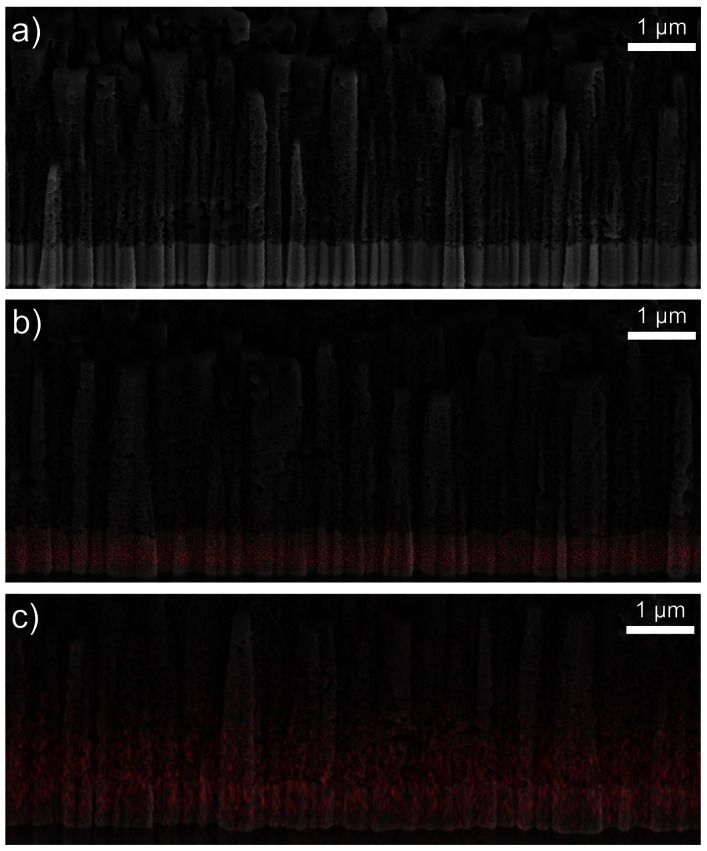
FIB image a) of the vertical DSC cross section; and with superimposed Sn EDX signal at b) (350°C) and c) (550°C).

**Figure 10:**
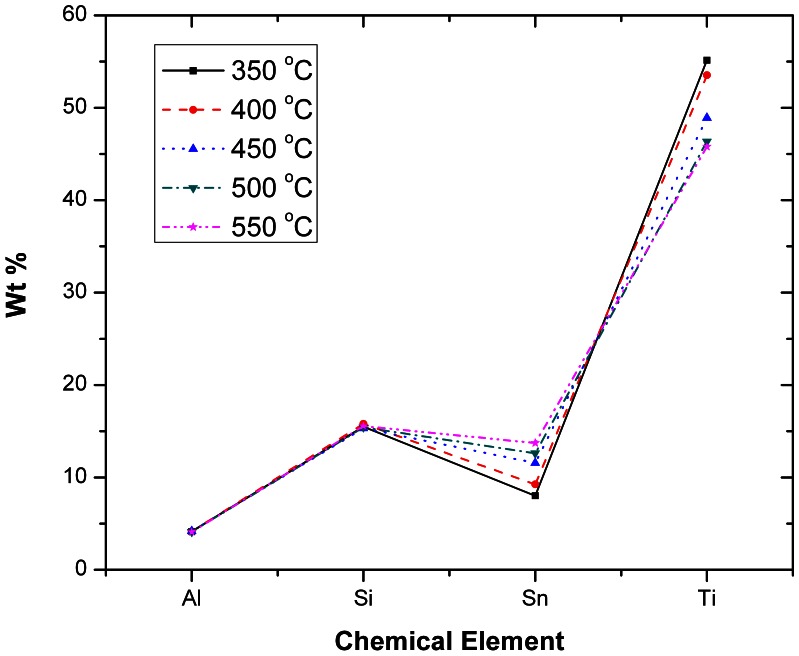
The EDX analysis (recorded for the areas in Figure 7 for each temperature) quantifying the Sn diffusion increase when increasing the sintering temperature from 350°C to 550°C.

For the spatial diffusion studies, the TiO_2_ active layer was not subject to any TiCl_4_ treatments or ruthenium dye adsorption before the shown EDX mapping. By using EDX we were able to achieve a sufficiently high spatial resolution analysis of the solar cell layers as shown in Figure 6b. It is important to note that from the Monte Carlo simulations shown in Figure 8 **,** it can be concluded that the spatial broadening of the EDX mapping is approx. 150 nm, which is smaller by an order of magnitude than the experimentally identified total Sn diffusion that was measured to be at 2 µm and this offers enough resolution. Furthermore, EDX measurements performed for samples sintered at higher temperatures and thus higher TiO_2_ densities due to the shrinking effect, show an increased Sn diffusion into the TiO_2_, compared to lower sintering temperatures, which is an effect that cannot be explained by the spatial broadening of the EDX mapping (i.e. it is clearly not an artefact). The EDX analysis for photoanodes sintered at 350°C (Figure 7a) confirmed that the Sn (FTO - red area) diffusion into the TiO_2_ starts already at comparably low temperatures. When investigating samples sintered at 400°C –550°C (Figure 7 b-e), EDX confirmed that the diffusion levels were consequentially increased proportional to the temperature. From 500°C on (Figure 7d), EDX mapping demonstrates a pronounced spatial extent of diffusion that was easily spatially resolved and quantified. Finally, when investigating samples sintered at 550°C (Figure 7e) the Sn diffusion levels were found to extend about 2 µm into the TiO_2_ mesoporous scaffold. The spatially resolved EDX analysis with the superimposed FIB imaging signals (Figure 9) are confirming the Sn diffusion results presented above, and come as reinforcement to the FTO diffusion enhancement with increasing temperatures as measured in the SEM-FIB analysis. From this it can be concluded that when increasing the sintering temperature from 350°C to 550°C, the FTO thickness decreases as a result to an increase in the Sn diffusion rate. The quantified effect of the above EDX analysis on the Sn diffusion for multiple samples is plotted in Figure 10. The major finding is that with the sintering temperature increase from 350°C to 550°C, the measured Sn level (in the FIB removed cuboid) increases from 8 wt% to 13.71 wt%, while the Ti percentage decreases accordingly from 55.14 wt% to 45.8 wt%. This indicates that with the sintering temperature increasing from 350°C to 550°C, the Sn is also increasingly diffusing onto the surface of the TiO_2_ layer within the removed cuboid, therefore the detected Sn signal increases, while the Ti signal decreases. The Al and Si percentages (coming from the glass) remain constant at 4.1 wt% and 15 wt% respectively as shown in the EDX analysis (Figure 10), which again reinforces the fact that the Sn diffusion is not an artefact of the EDX.

The next step of the analysis was to assemble the above photoanodes sintered at temperatures of 350°C –550°C into DSCs and to measure their efficiency. However, before assembling the DSCs, each FTO layer was tested regarding their behaviour to these sintering temperatures. The FTO conductivity was of particular importance in order to confirm that potential changes in the DSC efficiencies are not due to a change in FTO’s conductivity. The conductivity was measured for each sample, before and after sintering at temperatures up to 550°C, with a high accuracy Keithley 6514 electrometer. To determine the conductivity of the FTO, the resistance of the conductive layer was measured as the control parameter by attaching the electrometer probes to the extremities of each FTO glass slide (dimensions of 2×2 cm^2^). The high quality of the FTO slides was confirmed by measuring an initial average resistance of 23.1 Ω (±0.2%) for all the samples prior to sintering. The same measurements during the sintering also showed that the conductivity is significantly higher than the pre-sintering values, but it returns to the initial values during the cooling of the samples. Subsequent to sintering at temperatures of maximum 550°C, it was confirmed that the resistance remained constant on average at 23.1 Ω, with a difference of ±0.04–0.09% for each sample before and after the sintering. Since the resistance was constant, it was concluded that the conductivity of the FTO layer remained constant before and after sintering up to temperatures of 550°C. In order to get more results for supplemental temperatures, we have introduced in our efficiency analyses additional DSC photoanodes which have been sintered at temperatures of 425°C, 475°C and 525°C, respectively.

**Figure 11:**
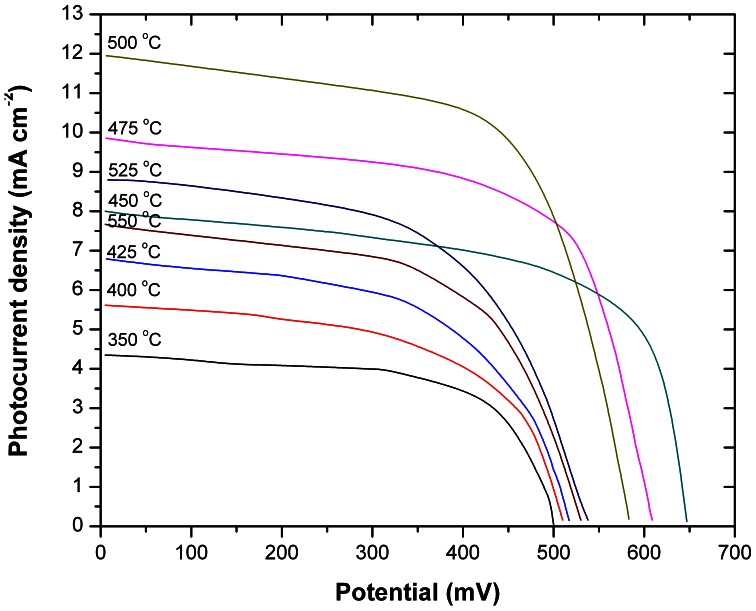
The J-V measurements for DSCs fabricated at a sintering temperature between 350°C –550°C.

Photocurrent and voltage measurements under simulated 1 sun AM 1.5 irradiance conditions were quantitatively recorded with a *Keithley 6514* electrometer. As can be seen from Figure 11, increasing the sintering temperature from 350°C to 500°C results in a significant increase of 273% in current density (J). Higher temperatures resulted in a decrease in current density. Regarding the voltage, increasing the sintering temperature from 350°C to 450°C improves the voltage by 129% – higher temperatures are detrimental to the DSC potential. Higher temperatures than 500°C lead to reduced current density and reduced voltage of the here prepared DSCs. The full results are shown in Table 1 that additionally includes the changes in the fill factor and in the overall DSC efficiency.

**Table 1:** DSC efficiencies recorded for various FTO doping levels achieved by modifying the sintering temperature from 350°C–550°C.

T (°C)	J_SC_ (mA cm^−2^)	V_OC_ (mV)	FF	η (%)
350	4.37	500	0.62	1.35
400	5	510	0.59	1.51
425	6.79	517	0.54	1.89
450	8	647	0.63	3.26
475	9.85	609	0.65	3.89
500	11.94	583	0.62	4.31
525	8.8	538	0.56	2.65
550	7.66	530	0.57	2.31

As reported in literature, high sintering temperatures cause an excellent removal of organic binders and solvents from within the TiO_2_ scaffold and improve the necking between the NPs [37]. Increasing the TiO_2_ sintering temperature from 350°C to 450°C, causes shrinking of the TiO_2_ thickness (Figure 4) translating into a reduced surface area but also resulting in a reduced number of charge-recombination sites, therefore the voltage increases. Furthermore, the increase in voltage can also be attributed to a negative shift (e.g. towards the dye LUMO level) in the flat band potential of the TiO_2_ semiconductor. This negative shift that causes the V_oc_ increase is attributed in our model to increasing Sn doping levels within the TiO_2_ film. However, when increasing the temperature above the found optimal point of 500°C and even though the above mentioned mechanisms (Sn doping, TiO_2_ layer shrinking etc.) continue to be produced as demonstrated above, the high sintering temperatures start to deteriorate the optimum interface conditions.

**Figure 12:**
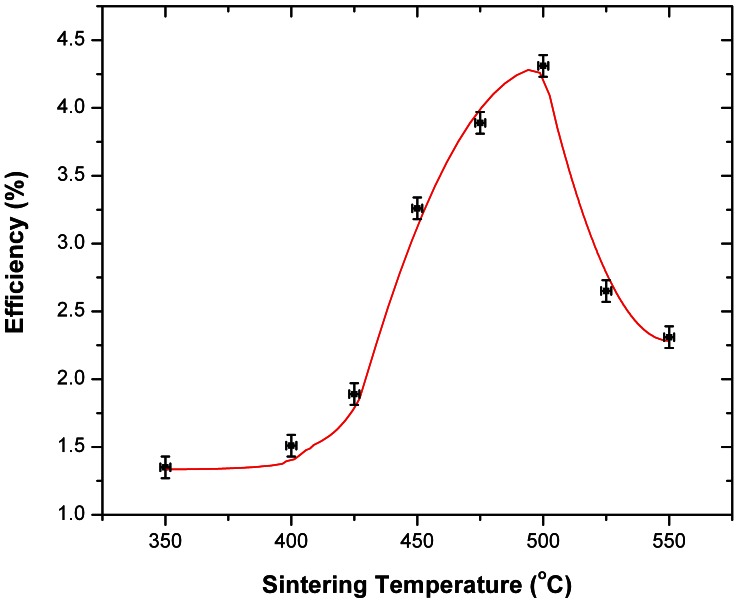
The DSC efficiency as a function of the sintering temperature (350°C –550°C).

Regarding the Sn diffusion effects on the J_SC_, as the temperature is increased from 350°C to 500°C, the NP necking improves and the TiO_2_ thickness decreases (Figure 4) and therefore the electrons will require a shorter diffusion length to reach the electrode, which results in the measured J_SC_ increase. However, although the temperature increase from 500°C to 525°C, and furthermore to 550°C, will cause a further shrinkage in the TiO_2_ thickness (Figure 4), the FTO diffusion process recorded here (Figure 10) will cause a drop in DSC efficiency for temperatures higher than 500°C (Figure 11). Finally, Figure 12 shows an overview of the measured DSC efficiencies for each particular sintering temperature based on the data presented in Table 1. The errors bars in Figure 12 represent the accuracy for the sintering temperature (±2°C) and for the efficiency measurements (±8%).

The here presented results confirm that in order to achieve a maximum DSC efficiency, effects produced by high levels of Sn doping into the TiO_2_ layer have to be taken into account and correlated with its side-effects on the V_OC_ and J_SC_.

### Conclusions

This study shows the effects of the sintering process between 350°C and 550°C on the FTO’s Sn migration into the TiO_2_ active layer using spatially resolved EDX analysis and FIB imaging. The FTO diffusion was proven not to negatively alter the V_OC_ for temperatures up to 450°C and the J_SC_ for temperatures below 500°C. Additionally we find for the here constructed solar cells, as described in the experimental section, that DSC efficiencies reach their maximum value at approximately a maximum sintering temperature of 500°C. As shown by J-V measurements, it can be concluded that sintering temperatures above this value are not recommended, as the DSC efficiency was measured here to decrease and the Sn diffusion rate increases furthermore. Future studies will look into the effects of prolonged sintering times for each temperature (in the range of 350°C –550°C) and the resulting potential DSC efficiency optimisation.

## References

[1] O’Regan B, Grätzel M (1991) A low-cost high effiiciency solar cell based on dye-sensitised colloidal titanium dioxide films. Nature 335: 737–739.

[2] Yella A, Lee HW, Tsao HN, Yi C, Chandiran AK, et al (2011) Porphyrin-Sensitized Solar Cells with Cobalt (II/III)–Based Redox Electrolyte Exceed 12 Percent Efficiency. Science 334: 629–634. 22053043 10.1126/science.1209688

[3] Gerischer H, Tributsch H (1968) Elektrochemische Untersuchungen zur spektralen Sensibilisierung von ZnO-Einkristallen. Ges. Phys.Chem. 72: 437–445.

[4] Tsubomura H, Matsumura M, Nomura Y, Amamiya T (1976) Dye sensitised zinc oxide: aqueous electrolyte: platinum photocell. Nature 261, 402–403.

[5] Dare-Edwards MP, Goodenough JB, Hamnet A, Seddon KR, Wright RD (1980) Sensitisation of semiconducting electrodes with ruthenium-based Dyes. Faraday Discuss. Chem. Soc. 70: 285–298.

[6] Duonghong D, Serpone N, Grätzel M (1984) Integrated systems for water cleavage by visible light; Sensitization of TiO_2_ particles by surface derivatization with ruthenium complexes. Helv. Chim. Acta 67: 1012–1018.

[7] Desilvestro J, Grätzel M, Kavan L, Moser JE, Augustynski J (1985) Highly efficient sensitization of titanium dioxide. J. Am. Chem. Soc. 107: 2988–2990.

[8] Namba S, Hishiki Y (1965) Color sensitization of zinc oxide with cyanine dyes. J. Phys. Chem. 69: 774–779.

[9] Vlachopoulos N, Liska P, Augustynski J, Grätzel M (1988) Very efficient visible light energy harvesting and conversion by spectral sensitization of high surface area polycrystalline titanium dioxide films. J. Am. Chem. Soc. 110: 1216–1220.

[10] Nazeeruddin MK, Kay A, Rodicio I, Humpbry-Baker R, Miiller E, et al (1993) Conversion of light to electricity by cis-X2bis(2,2′-bipyridyl-4,4′-dicarboxylate)ruthenium(II) charge-transfer sensitizers (X = Cl-, Br-, I-, CN-, and SCN-) on nanocrystalline titanium dioxide electrodes. J. Am. Chem. Soc. 115: 6382–6390.

[11] Nazeeruddin MK, Pechy P, Grätzel M (1997) Efficient panchromatic sensitization of nanocrystalline TiO_2_ films by a black dye based on a trithiocyanato-ruthenium complex. Chem. Commun. 1: 1705–1706.

[12] Green MA, Emery K, Hishikawa Y, Warta W (2009) Solar cell efficiency tables (version 34). Prog. Photovolt.: Res. Appl. 17: 85.

[13] Pechy P, Rotzinger F, Nazeeruddin MK, Kohle O, Zakeeruddin SM, et al (1995) Preparation of phosphonated polypyridyl ligands to anchor transition-metal complexes on oxide surfaces: application for the conversion of light to electricity with nanocrystalline TiO_2_ films. Chem. Commun. 1: 65–66.

[14] Tachibana Y, Haque SA, Mercer IP, Moser JE, Klug DR, et al (2001) Modulation of the rate of electron injection in dye sensitised nanocrystalline TiO_2_ films by externally applied bias. J. Phys. Chem. B 105: 7424–7431.

[15] Nazeeruddin MK, Humphry-Baker R, Liska P, Grätzel M (2003) Investigation of sensitizer adsorption and the influence of protons on current and voltage of a dye-sensitized nanocrystalline TiO_2_ solar cell. J. Phys. Chem. B 107: 8981–8987.

[16] Chiba Y, Islan A, Watanabe Y, Komiya R, Koida N, et al (2006) Dye-sensitized solar cells with conversion efficiency of 11.1%. Jpn. J. App. Phys. 45: 638–640.

[17] Shi D, Pootrakuchote N, Li R, Guo J, Wang Z, et al (2008) New efficiency records for stable dye-sensitized solar cells with low-volatility and ionic liquid electrolytes. J. Phys. Chem. C 112: 17046–17050.

[18] Ito S, Liska P, Comte P, Charvet R, Pechy P, et al (2005) Control of dark current in photoelectrochemical (TiO_2_/I^–^I_3_ ^−^) and dye-sensitized solar cells. Chem. Commun. 34: 4351–4353. 10.1039/b505718c 16113745

[19] Brown MD, Suteewong T, Kumar RS, D’Innocenzo V, Petrozza A, et al (2011) Plasmonic Dye-Sensitized Solar Cells Using Core-Shell Metal-Insulator. Nanoparticles Nano Lett. 11: 438–445. 10.1021/nl1031106 21194204

[20] Andrei C, Lestini E, Crosbie S, de Frein C, O’Reilly T, et al.. (2013) Plasmonic enhanced dye sensitised solar cells via chemical functionalisation of gold nanoparticles, Adv. En. Mat.: in press.

[21] Doyle G, Ashall B, Galvin M, Berndt M, Crosbie S, et al (2007) Mie scattering and surface plasmon based spectroscopy for the detection of nanoparticle-protein interaction. Appl. Phys. A. 89: 351–355.

[22] Williamson A, McClean E, Leipold D, Zerulla D, Runge E (2011) The design of efficient surface-plasmon-enhanced ultra-thin polymer-based solar cells. Appl. Phys. Lett. 99: 093307–093310.

[23] Kamat PV (2008) Quantum dot solar cells - Semiconductor nanocrystals as light harvesters. J. Phys. Chem. C 112: 18737–18753.

[24] Ito S, Murakami TN, Comte P, Liska P, Grätzel C, et al (2008) Fabrication of thin film dye sensitized solar cells with solar to electric power conversion efficiency over 10%. Thin Solid Films 516: 4613–4619.

[25] Grätzel M (2004) Conversion of sunlight to electric power by nanocrystalline dye-sensitised solar cells. J. of Photochem. and Photobiol. A 164: 3–14.

[26] Zhu K, Kopidakis N, Neale NR, van de Lagemaat J, Frank AJ (2006) Influence of surface area on charge transport and recombination in dye-sensitized TiO_2_ solar cells. J. Phys. Chem. B 110: 25174–25180. 10.1021/jp065284+ 17165961

[27] Lin J, Yu JC, Lo J, Lan SK (1999) Photocatalytic activity of rutile Ti_1−x_Sn_x_O_2_ solid solutions. J. Catal. 183: 368–372.

[28] Zheng SK, Wang TM, Hao WC, Shen R (2002) Improvement of photocatalytic activity of TiO_2_ thin film by Sn ion implantation. Vacuum 65: 155–159.

[29] Cao Y, Yang W, Zhang W, Liu G, Yue P (2004) Improved photocatalytic activity of Sn^4+^ doped TiO_2_ nanoparticulate films prepared by plasma-enhanced chemical vapor deposition. New J. Chem. 2: 218–222.

[30] Do YR, Lee W, Dwight K, Wold A (1994) The effect of WO_3_ on the photocatalytic activity of TiO_2_. J. Solid State Chem. 108: 198–201.

[31] Duan Y, Fu N, Liu Q, Fang Y, Zhou X, et al (2012) Sn-doped TiO_2_ photoanode for dye-sensitized solar cells. J. Phys. Chem. C 116: 8888–8893.

[32] Xin B, Ding D, Gao Y, Jin X, Fu H, et al (2009) Preparation of nanocrystalline Sn–TiO_2−X_ via a rapid and simple stannous chemical reducing route. Appl. Surf. Sci. 255: 5896–5901.

[33] Andrei C, O’Reilly T, Zerulla D (2010) A spatially resolved study on the Sn diffusion during the sintering process in the active layer of dye sensitised solar cells. Phys. Chem. Chem. Phys. 12: 7241–7245. 10.1039/c000072h 20495722

[34] Ottermann C, Otto J, Jeschkowski U, Anderson O, Fleming M, et al (1993) Stress of TiO_2_ thin films produced by different deposition techniques. Mater. Res. Soc. Symp. Proc. 308: 69.

[35] Smestad GP, Grätzel M (1998) Demonstrating Electron Transfer and Nanotechnology: A Natural Dye-Sensitized Nanocrystalline Energy Converter. J. Chem. Ed. 75: 752–756.

[36] Drouin XD, Couture AR, Joly D, Tastet X, Aimez V, et al (2007) A fast and easy-to-use modeling tool for scanning electron microscopy and microanalysis users. Scanning 29: 92–101. 17455283 10.1002/sca.20000

[37] Meen TH, Water W, Chen WR, Chao SM, Ji LW, et al (2009) Application of TiO_2_ nano-particles on the electrode of dye-sensitized solar cells. J. Phys. and Chem. of Sol. 70: 472–476.

